# Botany, Ethnomedicinal Uses, Biological Activities, Phytochemistry, and Technological Applications of *Morinda citrifolia* Plants

**DOI:** 10.3390/molecules30183831

**Published:** 2025-09-21

**Authors:** José Adão Carvalho Nascimento Júnior, Anamaria Mendonça Santos, Ana Maria Santos Oliveira, Cláudio Carvalho Santana Júnior, Mairim Russo Serafini, Jullyana de Souza Siqueira Quintans, Laurent Picot, Irwin Rose Alencar de Menezes, Lucindo José Quintans-Júnior

**Affiliations:** 1 Post-Graduate Program in Health Sciences, Federal University of Sergipe, Aracaju 49060-676, Sergipe, Brazil; adaocarv95@gmail.com (J.A.C.N.J.); anamariams@academico.ufs.br (A.M.S.); cl.junior@academico.ufs.br (C.C.S.J.); mairim@academico.ufs.br (M.R.S.); 2 Post-Graduate Program in Pharmaceutical Sciences, Federal University of Sergipe, São Cristóvão 49100-000, Sergipe, Brazil; anaoliveira.farmacia@gmail.com (A.M.S.O.); jullyanas@academico.ufs.br (J.d.S.S.Q.); 3 UMRi CNRS 7266 LIENSs, University of La Rochelle, 17042 La Rochelle, France; laurent.picot@univ-lr.fr; 4 Post-Graduate Program in Chemical Biology, Regional University of Cariri—URCA, Crato 63105-000, Ceará, Brazil

**Keywords:** antioxidant activity, antimicrobial activity, flavonoids, noni, traditional medicine

## Abstract

*Morinda citrifolia* L., known as noni, is a tropical plant belonging to the *Rubiaceae* family and is widely used in traditional medicine for its therapeutic properties. This review compiles botanical, ethnomedicinal, phytochemical, and pharmacological information about the species, as well as its technological applications. Studies indicate that various parts of the plant, such as the fruits, leaves, seeds, and roots, contain bioactive compounds such as flavonoids, iridoids, alkaloids, and phenolic acids. These compounds are associated with antioxidant, antimicrobial, anti-inflammatory, wound-healing, gastroprotective, antidiabetic, and hypocholesterolemic activities. Among these, antioxidant activity is particularly notable, with different extracts and isolated compounds demonstrating potent free radical scavenging capabilities. Additionally, the antimicrobial potential against resistant bacteria, healing effects observed in animal models, and immunomodulatory properties further support the traditional therapeutic use of noni. Moreover, the plant shows promise for applications in the formulation of pharmaceutical, cosmetic, and nutraceutical products. Although evidence of its beneficial effects is growing, further clinical studies and standardization of extracts are necessary to ensure its safe and effective use in medical practice.

## 1. Introduction

*Morinda citrifolia* L., commonly known as noni, is a small evergreen tree native to Southeast Asia and Australia. It is now widely cultivated across tropical and subtropical regions, including the Pacific Islands, Puerto Rico, the Dominican Republic, and Brazil, particularly in the Central-West and Northeast regions. This species has attracted considerable attention due to its extensive use in traditional medicine, as well as its potential therapeutic and industrial applications [1,2].

Historically, *M. citrifolia* has been utilized by indigenous populations as a natural remedy for a variety of ailments, including arthritis, headaches, burns, tuberculosis, diabetes, and hypertension [1]. These ethnomedicinal applications have spurred scientific investigations into the plant’s biological activities and phytochemical composition. Numerous studies have identified a diverse array of bioactive compounds in its various parts, particularly in the leaves, including flavonoids, saponins, steroids, cardiac glycosides, tannins, terpenes, and alkaloids. Many of these compounds are associated with antioxidant, anti-inflammatory, antimicrobial, and other pharmacological effects [3].

This literature review aims to provide a comprehensive overview of *M. citrifolia,* encompassing its botanical characteristics, ethnomedicinal uses, biological activities, phytochemical profile, and current technological applications.

## 2. Review

### 2.1. Botanical Attributes

**Figure 1:**
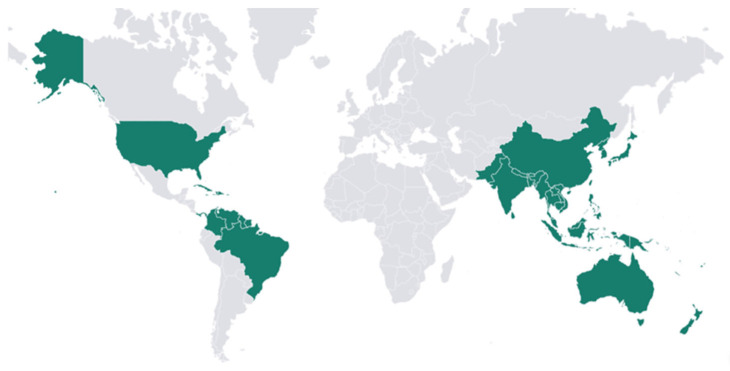
Geographical distribution of *Morinda citrifolia* L. around the world. **Source of the figure:** Prepared by the authors using Visme software 6.8.1.

*M. citrifolia* L. is a small tree, commonly known as noni, that belongs to the *Rubiaceae* family, which is recognized for its ecological adaptability. This shrub is cultivated in nearly all tropical and subtropical zones worldwide. Its adaptation allows the species to tolerate a diverse type of soil. This explains its natural distribution in Southeast Asia, the Pacific Islands, and tropical Australia. It also accounts for its successful endurance when it is brought to other parts of the world, including Brazil, the Caribbean, and Central and Northern South America (Figure 1) [4]. Vasconcelos et al. (2021), who used noni from Brazil, describe that noni plants flourish and bear fruit throughout the whole year, with peak production typically during the months of heavy rain, generally in the spring and summer, in the countries in which the plant is found [5].

The geographical origin of cultivation may affect the content of the fruit in terms of protein, fat, carbohydrate content, and its pH level, as described by Ratanavalachai and Thitiorul (2008), who compared the composition profile of noni fruit borne in Thailand and French Polynesia [6]. These factors directly influence the production and availability of active secondary metabolites in plants, including polyphenolics, organic acids, and alkaloids, which, in turn, impact the biological activities exhibited by noni. Furthermore, Clafshenkel et al. (2012) use Tahitian noni fruit and mention iridoid as one class, among others, of active phytochemical components with altered concentrations based on the region of harvesting and parts of the plant used at the formulation [7]. Beyond that, different metabolite profiles are also defined by the ripeness of the fruit, which is indicated by size, shape, and color. This indicates that noni fruits that are rounded, have a pale yellowish hue, and are ripe possess higher levels of phenolics and polysaccharides [8].

**Figure 2:**
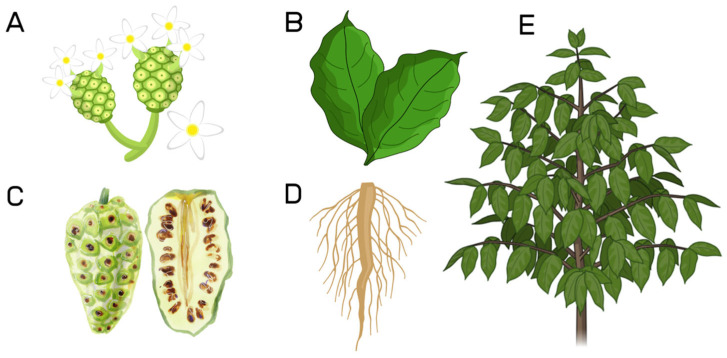
General aspect of *Morinda citrifolia* L. (**A**) Flower and floral buds; (**B**) Leaves; (**C**) Fruit; (**D**) Roots; (**E**) Stems with leaves. **Source of the figure:** Prepared by the authors using Canva software 1.116.0.

*M. citrifolia* possesses an angular stem with leaves that are broadly elliptic, large, glabrous, and shine with very prominent veins, which gives the leaf a robust appearance [9]. The fruit is oblong and green in colour, with a lumpy surface covered by prominent black dots in polygonal-shaped sections, and has an edible pulp yellowish white in color, with a soft and fleshy texture. Noni’s texture is achieved through its ratio of wet and dry material, possessing mostly water but also fibers and soluble solids [10,11]. As the fruit matures, the pulp undergoes a color transition, shifting from green to a pale yellowish hue. It emits a distinctive odor, often described as a mild cheesy fragrance, with a very bitter taste (Figure 2) [12]. The roots of this shrub exhibit a taproot structure, indicative of efficient anchorage and deep soil penetration, and, as demonstrated by Baque et al. (2014), serve as a focal point for exploring larger-scale production of bioactive compounds [13].

### 2.2. Ethnomedicinal Uses

Noni, with all its different parts (fruit, leaf, bark, root, flower, and seed), has been used in traditional medicine for treatment and prevention of a variety of illnesses in its native countries, which is a reflection of both the ecological conditions of specific locations and the accumulated traditional knowledge transmitted through generations. Noni is integrated into folk medicine as a multipurpose therapy; it shares the unifying theme of a versatile remedy for ailments worldwide [14]. Traditional use of noni fruit includes the prevention of indigestion, nausea, vomiting, and constipation, as well as its antibacterial and antifungal effects [15]. Noni’s leaves are used to promote healing of wounds, ulcers, erythema, and inflamed areas and to modulate immune responses, which are of great pathological relevance in the development of diabetes, cancer, arthritis, cardiovascular, and autoimmune diseases [16,17]. These ethnopharmacological claims are currently being substantiated by preclinical and experimental research [18,19].

In Asia, the fruit is often consumed fresh or as decoctions, while the leaves are applied topically for skin ailments and wounds in order to treat infections [20]. Due to its ability to alleviate joint pain, fatigue, and inflammatory disorders, it is also described as a vitality-enhancing tonic, especially in China. In the Pacific Islands, particularly in Tahiti, noni fruit juice is consumed for general well-being and as a protective tonic against lifestyle-related diseases. Similarly, Fijian and Cook Islands’ communities traditionally use the fruit and leaves for wound healing, infections, and gastrointestinal disturbances [21]. Beyond Asia and the Pacific, ethnomedicinal practices further highlight noni’s versatility. In the Caribbean, the plant is referred to as the “painkiller bush”, emphasizing its role in the relief of pain [22]. In Polynesia, traditional healers made extensive use of every part of the plant: the fruit for gastrointestinal and respiratory conditions, the leaves for topical poultices against inflammation, and the roots for systemic ailments [23]. In Hawaii, and more recently in Europe, the fermented juice became central to healing practices, administered for treating various physiological disorders [24].

Therefore, *M. citrifolia* presents promising therapeutic use considering its diverse spectrum of properties. Its traditional uses are increasingly supported by experimental evidence, particularly for conditions such as inflammation, oxidative stress modulation, and bacterial infections. However, future research and clinical trials are still essential to continue to validate these applications and to develop safe and standardized formulations for their use in medicine.

### 2.3. Phytochemistry

Understanding the phytochemistry of medicinal plants is essential to determine the compounds with therapeutic activities for the development of the pharmaceutical industry and to promote the process of extracting components more efficiently [25,26,27]. In this context, it is important to evaluate the medicinal properties of *M. citrifolia*. Noni leaves have flavonoids, triterpenoids, alkaloids, coumarins, anthraquinones, saponins, tannins, carotenoids, organic acids, and reducing agents; its fruits have a large amount of polyphenols and antioxidant agents, and its roots have a chemical composition marked by the presence of anthraquinones (Table 1) [28,29,30].

**Figure 3:**
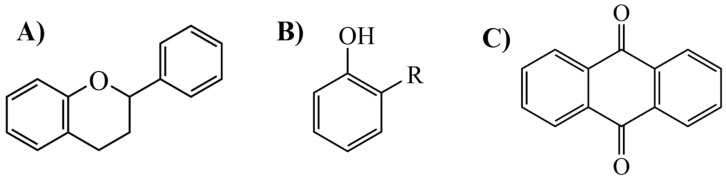
Basic chemical structure: (**A**) Flavonoid; (**B**) Phenolic compound; (**C**) Anthraquinones. **Source of the figure:** Prepared by the authors using ChemDraw Professional software 16.0.

Flavonoids, which are compounds with two aromatic rings and a pyran ring, exhibit antioxidant, anti-inflammatory, and antimicrobial activities, which are linked to their chemical structure, particularly the number and position of hydroxyl groups or glycosylation [31,32,33]. Phenolic compounds are characterized by the presence of an aromatic ring bearing one or more hydroxyl groups, whose antioxidant and anticancer activity is associated with factors such as the presence of conjugated double bonds and the degree of polymerization [34]. Anthraquinones have two benzene rings joined by a central quinone ring, known as the 9,10-anthracenedione core, where their therapeutic activity is linked to the presence and position of hydroxyl groups, polarity, and type of substituents, interfering with antioxidant and cytotoxic action (Figure 3) [35,36].

Among the predominant compounds of *M. citrifolia*, anthraquinones and xanthones were identified in studies conducted by Su et al. (2004), Siddiqui et al. (2005), and Shen et al. (2021) [30,37,38]. Based on the extracts produced, the researchers confirmed the presence of the anthraquinones morindicone (9-hydroxy-2-methoxy-4-methyl-3,10-anthracenedione), 1-hydroxy-2-methylanthraquinone, 2-hydroxymethylanthraquinone, 1,3,5-trihydroxy-2-methoxy-6-(methoxymethyl)-9,10-anthracenedione, and 3-hydroxy-1,2,4-trimethoxy-6-methylanthraquinone; in addition, the xanthones morinthone (4-methoxy-3-heptadecylxanthone) and plocamanone C were also identified [30,37,38].

However, a notable gap remains in the comparative quantification of anthraquinones in different parts of *M. citrifolia*. Although precise standardized values are scarce, several studies consistently indicate that anthraquinones are predominantly concentrated in the roots, while the stem and leaves contain moderate to low levels of anthraquinones, and the fruits exhibit only small amounts of well-defined compounds. To illustrate this distribution, Table 2 summarizes the relative abundance of anthraquinones across different plant parts, highlighting the root as the most pharmacologically relevant source for therapeutic development [39,40].

Glycosylated compounds make up most of the phytochemical profile of noni. Among them, iridoid glycosides are prominent, such as asperuloside, asperulosidic acid, geniposidic acid, and deacetylasperulosidic acid, identified from studies by Su et al. (2005) and Akihisa et al. (2012) [37,41]. Although there are not many studies evaluating this quantitatively, there is a difference in the concentration of iridoid glycosides depending on the ripeness of the fruit, such as asperuloside, which is not found in ripe noni fruit [42]. Nevertheless, other glycoside-derived compounds were also identified by Akihisa et al. (2012), with the presence of nonioside D, F, and C. In addition, nonioside A, 3-methylbut-3-en-1-yl-β-D-glucopyranoside was identified by De La Cruz-Sanchez et al. (2019), who extracted *M. citrifolia* seeds and found γ-lactones, such as 5-butyloxolan-2-one and 5-hexyloxolan-2-one [41,43]. Furthermore, in studies by Su et al. (2005) and Akihisa et al. (2012), nucleosides and alcohols such as D-mannitol, as well as vanillic acid and scopoletin, belonging to the phenolite compounds, were identified [37,41].

*M. citrifolia* also has several lipophilic compounds, belonging to the classes of fatty acid esters, sterols, triterpenes, and alkenes. In this context, the research by De La Cruz-Sanchez et al. (2019) identified methylated fatty acid esters such as methyl linoleate, methyl palmitate, methyl oleate, and methyl stearate, and (E)-octacos-2-ene, an unsaturated hydrocarbon, in the seed extracts [43]. Regarding sterols, Akihisa et al. (2012) identified them in the form of β-sitosterol, stigmasterol, and β-sitosterol 3-O-β-D-glucopyranoside, as well as other lipophilic compounds, such as ursolic acid and oleanolic acid [41].

Finally, other less predominant secondary metabolites were observed. Among them are terpenoids, such as (+)-austrosene, (6S)-2-methyl-6-(4-formylphenyl)-2-hepten-4-one, and asperterpenoid A, documented by Zhao et al. (2022); alkaloids, such as borreriagenin, and the neolignan americanin A, were identified by Su et al. (2024) [44].

Furthermore, different extraction methods and geographic location, as well as the nature of the solvent, can also influence the concentration of active secondary metabolites in the extract, which can increase or decrease the therapeutic action of noni. For example, Deng et al. (2011) reported that geographical factors exert a significant influence on the phytochemical composition of *M. citrifolia* fruits, with iridoid concentrations ranging from 13.8 to 42.9 mg/g, depending on the growing region [45]. In a related study, Pongnaravane et al. (2006) demonstrated that pressurized hot water extraction and Soxhlet extraction produced extracts with superior antioxidant activity compared to conventional methods such as maceration [46]. Similarly, Hemwimol et al. (2006) [47] highlighted that the efficiency of anthraquinone recovery through ultrasound-assisted extraction was strongly dependent on the solvent employed, with acetone providing the highest recovery, followed by acetonitrile, methanol, and finally ethanol. Collectively, these findings highlight that both environmental conditions and extraction methodologies play essential roles in determining the antioxidant potential of *M. citrifolia* extracts [47].

### 2.4. Biological and Pharmacological Activities

#### 2.4.1. Antioxidant Activity

Antioxidant activity refers to the ability of a substance to inhibit or delay the oxidation of other molecules, preventing damage caused by reactive oxygen species (ROS) and reactive nitrogen species (RNS). These species can oxidize essential cellular components such as lipids, proteins, carbohydrates, and nucleic acids, resulting in oxidative stress, which is related to aging and various diseases [48]. In this sense, antioxidant activity plays a fundamental role in maintaining cellular homeostasis and protecting against degenerative processes. In practical terms, it represents the ability of natural or synthetic compounds to neutralize free radicals before they cause irreversible damage to cells. This ability is particularly relevant in tissues with a high metabolic rate or vulnerable to the accumulation of reactive species, such as the brain, where oxidative stress is implicated in the etiology of neurodegenerative diseases such as Alzheimer’s [49].

Antioxidant activity, when analyzed under a molecular and functional approach, should be understood as an integrative parameter, with the ability to predict the therapeutic potential of bioactive compounds, representing an essential mechanism of biological defense, whose stimulation or supplementation, through natural compounds, contributes significantly to health promotion and the prevention of diseases associated with oxidative stress [50]. Antioxidant activity has great relevance in the biological and pharmacological context due to its ability to modulate the oxidation reaction in the body and in experimental systems [51]. In this sense, the half maximal inhibitory concentration (IC50) is widely employed as a standard parameter to quantify antioxidant potential, since it represents the concentration of a compound required to inhibit 50% of oxidative activity, being a reliable indicator of potency in comparative studies [52].

Phytochemical prospecting of the *M. citrifolia* revealed the presence of several bioactive secondary metabolites. The analysis allowed the identification and isolation of lignans, including americanin, which is recognized for its structure that confers antioxidant and anti-inflammatory properties, and two additional lignans not yet named, in addition to scopoletin, which are secondary metabolites derived from the life of shikimate. In addition, glycosides were identified, classified as compounds A-D, which may influence their solubility, transport, and bioavailability in the body [37,53].

Biologically, antioxidants participate in maintaining the cellular redox balance, regulating processes such as intracellular signaling, gene expression, and the structural integrity of biomolecules such as lipids, proteins, and nucleic acids. From a pharmacological point of view, these compounds act as functional agents in therapeutic formulations, contributing to the chemical stability of active ingredients, protecting them against oxidative degradation, and enhancing their bioavailability and efficacy [54]. In this context, the search for natural sources of antioxidants has intensified, especially due to the need for safe and bioactive alternatives to antioxidants (Table 3 and Table 4).

The study conducted by Sevalho et al. (2017) reinforces this trend by highlighting the high antioxidant potential of *M. citrifolia* extracts, especially the ethyl acetate extract of the seeds, using the Thin-layer chromatography (TLC) with 1,1-diphenyl-2-picrylhydrazyl (DPPH), which is a bioautographic technique used to separate the compounds in an extract and reveal the compounds with significant free radical scavenging activity, even in the absence of classical phenolics or flavonoids [55]. In parallel with its stabilizing function in pharmaceutical formulations, antioxidant activity also plays a profound physiological role by protecting cells against the harmful effects of free radicals, which can trigger highly damaging chain reactions.

This capacity was evidenced in the study by Ezenwaka et al. (2018) [56], which compared the antioxidant potential of the methanolic extracts of the fruits of *M. citrifolia*, demonstrating significant action in the neutralization of various radicals such as superoxide, hydroxyl, and nitric oxide in a dose-dependent pattern. Notably, the *Morinda* extract showed better performance against the superoxide radical, surpassing vitamin C, which reinforces its effectiveness as a reducing agent in intense oxidative environments [56]. In addition, many natural antioxidants demonstrate multiple bioactivities, including anti-inflammatory, metal chelating, and enzyme modulating properties, which make them promising targets for the development of new pharmaceutical and nutraceutical products [57]. The enhanced antioxidant capacity of *M. citrifolia* in comparison to vitamin C may be attributed to the nature of the solvent employed during the extraction process. As reported by Jelena Mitrovic et al. (2024) [58], extracts obtained using polar solvents, particularly methanol, demonstrate superior antioxidant potential. This phenomenon is primarily associated with the increased efficiency of polar solvents in extracting phenolic compounds and flavonoids, which are bioactive constituents well recognized for their significant contribution to antioxidant activity [58].

The ethanolic extract of *M. citrifolia* Linn. leaves showed significant antioxidant activity in in vitro tests using the DPPH method, which is a stable free radical widely used as a marker for antioxidant activity tests, due to its intense color and reactivity with hydrogen or electron donors. In the present study, Rodrigues et al. (2017) identified an inhibitory concentration (IC 50) value of 4.27 ± 0.004 g/L, with a linear coefficient of determination of 0.9954, indicating a high capacity of the extract to neutralize free radicals, an action that was mainly attributed to the presence of flavonoids detected in the phytochemical prospection of the leaf, which are known for their antioxidant properties [59].

The antioxidant activity of *M. citrifolia* has been highlighted as a plant of great relevance due to its expressiveness, as it is widely associated with a variety of pharmacological and biological effects. According to Choi et al. (2020) [60], in particular, the aqueous extract of *M. citrifolia* fruit showed high antioxidant activity in a cellular model; through DPPH assays, the extract showed efficacy of up to 97%, even surpassing ascorbic acid, which is used as a positive control. Treatment with the extract resulted in a significant reduction in inflammatory mediators, such as interleukin-6 (IL-6), which acts as a pleiotropic molecule that promotes lymphocyte differentiation, and tumor necrosis factor alpha (TNF-α), which is a potent inducer of inflammation, promoting the expression of adhesion molecules, chemokines, and metalloproteinases, contributing to tissue damage and cell apoptosis [61]. Beyond macrophages, other studies report immunomodulatory actions involving adaptive immune cells. For instance, polysaccharide-rich fractions of *M. citrifolia* were shown to enhance CD8^+^ T lymphocyte activity and stimulate NK cell proliferation and cytotoxicity, suggesting broader immunostimulatory effects than previously recognized. This highlights the potential of *M. citrifolia* not only in innate immune modulation but also in adaptive responses, which could be therapeutically relevant in infection control and cancer immunotherapy [60,62].

From a pharmacological point of view, *M. citrifolia* L. has great potential, which is largely justified by its rich phytochemical composition and potent antioxidant activity. According to Fontes et al. (2023) [63], this matrix has been shown to have high levels of phenolic compounds (7486.38 µg GAE/g) and flavonoids (385.57 µg QE/g), as well as high levels of vitamin C (336.62 µg QE/g) and carotenoids. The high antioxidant capacity was confirmed by in vitro Ferric Reducing Antioxidant Power (FRAP) and 2,2′-Azino-bis(3-ethylbenzothiazoline-6-sulfonic acid) (ABTS) methods, which are methods used to assess the in vitro antioxidant activity of compounds, with values of up to 467970.40 mmol/100g of ascorbic acid equivalence standing out, showing the efficiency of the freeze-dried pulp in neutralizing free radicals [63].

At the same time, according to Semwal et al. (2021), rutin, which is a major flavonoid found in the pulp and bark of noni, has a great ability to inhibit the production of pro-inflammatory cytokines such as TNF-α and IL-6 due to its potent antioxidant action, with the ability to stabilize reactive oxygen species and protect cell membranes against lipid peroxidation [64]. In addition, Wang et al. (2022), demonstrated the action of vanillin in experimental models, which in turn acted as an antioxidant agent, reducing cell damage mediated by free radicals, which in turn stands out as a phenolic compound, acting in the neutralization of reactive oxygen species and in the reduction of cellular oxidative damage; its protective effects are attributed to the ability to modulate biochemical pathways involved in oxidative stress, conferring stability to cellular structures [65].

#### 2.4.2. Antimicrobial Activity

Antimicrobial activity refers to the ability of a natural or synthetic compound to inhibit the growth of or eliminate pathogenic microorganisms such as bacteria, fungi, viruses, and parasites [66]. According to Zhang et al. (2021), the importance of this property lies in its essential role in the prevention and treatment of infections, especially in the face of the growing problem of antimicrobial resistance, which renders many conventional antibiotics ineffective [67]. Corroborating this relevance, Dian et al. (2020) [68] showed that the combination of organic acids such as benzoic, formic, and fumaric acid alone had a significant inhibitory effect against strains of *Escherichia coli* and *Salmonella typhi* in an in vitro test, with the compounds tested having a minimum inhibitory concentration (MIC) of 50% for *E. coli* and 100% for *Salmonella typhi. coli* and 100% for Salmonella, with a total reduction in microbial viability after 13 to 14 h of exposure, depending on the formulation. Results such as these reinforce that natural substances such as phenolic extracts and organic acids can represent effective alternatives to conventional antimicrobials [68]. Regarding antiviral potential, evidence remains less developed, but several phytochemicals from *M. citrifolia*, including iridoids, flavonoids, terpenoids, and lignans, exhibit structural features that may interact with viral enzymes and inhibit key steps of viral replication. Notably, bisabolane-type sesquiterpenes isolated from *M. citrifolia* demonstrated significant inhibitory activity against HIV-1 reverse transcriptase, with median effective concentration (EC_50_) values ranging from 0.16 to 6.29 μM. These findings suggest that beyond theoretical interactions, *M. citrifolia*-derived compounds hold antiviral potential, opening promising avenues for deeper investigation into their use as scaffolds for novel antiviral drug development [44].

The antimicrobial effect is attributed to the presence of bioactive compounds such as flavonoids, alkaloids, lignins, coumarins, and antimicrobial peptides (Table 5 and Table 6). Research has shown that the methanolic extracts of *M. citrifolia* seeds have significant activity against clinical strains of methicillin-resistant *Staphylococcus aureus* (MRSA), with MICs ranging from 16 to 200 μg/mL. Isolated compounds such as americanin A and scopoletin have been identified as responsible for this activity, suggesting that they have great potential as natural antibacterial agents [69].

Medrano-Colmenares et al. (2024) [70] showed that the methanolic extract of the bark, pulp, and seeds of *M. citrifolia* has fungistatic and fungicidal effects, inhibiting fungal growth and killing the fungus *Candida albicans*. The seed extract showed high sensitivity against this type of fungus with a minimum inhibitory concentration (MIC) of 1366.25 mg/mL [70]. In addition, Kaul et al. (2023) incorporated *M. citrifolia* extracts in the form of silver nanoparticles (AgNPs) into dental prosthesis base resin, observing a significant reduction in *Candida albicans* adhesion on surfaces treated with 0.1% AgNPs from the plant, indicating its antifungal and antimicrobial potential applied to clinical biomaterials [71]. In studies carried out by De La Cruz-Sánchez et al. (2019) [43], the methanolic extract of *M. citrifolia* seeds showed significant activity against multidrug-resistant clinical strains of *Staphylococcus aureus*, *S. epidermids, and S. haemolyticus*. The MIC of the crude extract was 16 mg/mL, with the methanolic extract being the most potent among the solvents tested, with purified fractions containing scopoletin and americanin showing MICs between 25 and 100 µg/mL, indicating high efficacy even at low concentration levels [43].

Obeng-Boateng et al. (2024) [72] observed the antibacterial activity of *M. citrifolia* extracts against eight pathogenic bacterial species. For this experiment, different parts of the plant were used, such as the root, leaf, and fruit in different forms (fresh, dried, and fermented). The extracts were prepared with distilled water and ethanol in concentrations of 60%, 80%, and 100%, and their efficacy was assessed using the agar diffusion method, with ciprofloxacin as a positive control. The results showed that, in general, the extracts made with pure ethanol were more effective in inhibiting the bacteria, showing larger zones of inhibition than the extracts made with water alone. The root extract prepared with 80% ethanol had the best results, forming an average inhibition zone of 21.33 ± 1.80 mm against the *Campylobacter* spp. bacteria. In addition, the *Enterococcus faecium* and *Bacillus cereus* bacteria were very sensitive to all the extracts tested, while *Higella* spp. and *Klebsiella* spp. were more resistant [72].

Complementing these findings, Oliveira et al. (2022) [73] investigated the antimicrobial potential of endophytic fungi isolated from *M. citrifolia* against *Xanthomonas axonopodis*, which is the causative agent of bacterial blotch in passion fruit trees. The fungus *Guignardia mangiferae* produced two compounds, Sydowinol and Sydowimim A, which showed bactericidal activity up to the lowest concentration tested (3.125 µg/mL), revealing the potential of *M. citrifolia* as a source of agricultural antimicrobial agents of natural origin [73].

#### 2.4.3. Healing and Anti-Inflammatory Activity

In the study conducted by Ly et al. (2020) [28], it was observed that a 70% ethanolic extract of *M. citrifolia* leaves had a significant healing effect on an excisional wound model in mice. The animals treated topically with 1% and 5% concentrations of the extract demonstrated wound contractions of 92.45% and 87.18%, respectively, after 11 days, while the control group exhibited only 54.46% contraction. Furthermore, histological analysis revealed that the groups treated with the *M. citrifolia* leaf extract exhibited normal regeneration of the epidermis and reorganization of the skin tissue, accompanied by fibroblast proliferation and a reduction in the inflammatory infiltrate [28].

Although most of the evidence on the healing potential of *M. citrifolia* comes from animal models, some preliminary clinical studies also show benefits in humans. A relevant example is described by West (2018) [74] in an open-label prospective study conducted with 30 children with burn injuries, aged 2 to 9 years, admitted to a hospital in Bolivia. In this trial, patients received 30 mL of noni juice twice a day for up to 30 days in combination with conventional treatment. The results indicated faster granulation, improved quality of the granulation tissue formed, and reduced hospitalization time when compared to the usual recovery pattern. Although there are methodological limitations, such as the low number of participants, the results reinforce the hypothesis that noni may have beneficial effects on healing in humans, in line with the mechanisms already observed in experimental studies [74].

The study conducted by researchers Kim, Seong, and Choung (2020) [53] investigated the effects of treatment with fermented *M. citrifolia* on skin lesions similar to atopic dermatitis in mice. Oral administration of fermented *M.a citrifolia* at doses of 250, 500, and 1000 mg/kg significantly reduced lesions and symptoms associated with atopic dermatitis, including ear thickness, dermatitis score, the number of infiltrated mast cells and eosinophils, as well as scratching behavior, when compared to the control group. Serum IgE levels in the treatment group decreased approximately twofold, from 2400 µg/mL to 1270 µg/mL, relative to the control group. Furthermore, the treatment reduced histamine levels and inhibited the expression of inflammatory cytokines such as IL-4, IL-17, and IL-22, while restoring the expression of proteins important for the skin barrier, such as filaggrin, loricrin, and involucrin. Thus, according to the authors, the beneficial effects of fermented noni on lesions similar to atopic dermatitis result from synergistic actions of multiple compounds in the extract, which simultaneously modulate Th2-, Th17-, and Th22-mediated inflammation and promote skin barrier restoration [53].

Alkausar et al. (2024) [75] observed that a gel formulation containing extracts of noni and *Aloe vera* significantly enhanced the healing of burns in white mice (*Mus musculus*). The gel, which contained 5% noni and 0.5% *Aloe vera*, demonstrated results comparable to a commercial gel. After 15 days, the average wound diameter in the treatment group was 4.04 cm, significantly smaller than the untreated group, which measured 6.53 cm. This finding suggests that the combination of these plants acts synergistically, attributed to active compounds such as mannose-6-phosphate from *Aloe vera*, which stimulates fibroblast and collagen synthesis, and antioxidants from noni, including flavonoids, tannins, triterpenes, and polyphenols, which exhibit anti-inflammatory and antimicrobial properties [75].

In addition, Sousa et al. (2018) [76] used an excision wound model in streptozotocin-induced diabetic rats to investigate the healing effects of noni fruit juice. The animals were divided, and a group of diabetics was treated with noni juice at a dosage of 100 mL/kg, administered via drinking water for 10 days. The results indicated that the group treated with noni juice experienced a 73% reduction in wound area, which was significantly higher than the 63% reduction observed in the diabetic control group. Furthermore, there was a notable increase in granulation tissue weight and hydroxyproline content, suggesting enhanced collagen deposition. Although the study primarily focused on wound healing, the authors proposed that these benefits may be linked to the anti-inflammatory properties of noni, which contribute to reducing the inflammation and oxidative stress that typically impede healing in diabetic wounds [76]. Compared to standard therapies such as metformin, which inhibits hepatic glucose production and improves insulin sensitivity in peripheral tissues, noni juice stands out for its ability to modulate inflammation and accelerate tissue healing [77,78]. Thus, while metformin has a direct metabolic effect on controlling hyperglycemia, noni has a complementary effect, contributing to the reduction of secondary complications of diabetes, such as delayed wound healing [77,78].

West et al. (2009) [17] found in their studies that the ethanolic extracts and juice of *M. citrifolia* leaves exhibit significant topical anti-inflammatory activity. In a model of erythema induced by UVB radiation in volunteers, the application of the extracts increased the minimum dose required to induce erythema by nearly 3.5 times compared to untreated skin. This effect was comparable to that of formulations containing 8% homosalate, highlighting the photoprotective and soothing potential of noni extracts. Additionally, in vitro tests demonstrated that the ethanolic extract of the leaves inhibited binding to the histamine H-1 receptor by 57%, suggesting a possible mechanism of action related to the modulation of histamine-mediated inflammatory pathways [17].

#### 2.4.4. Gastroprotective Activity

The gastrointestinal effects of the polyphenols found in the fruit of *M. citrifolia* were investigated in a study conducted by Chen et al. (2024) [79]. Dried noni fruit powder was utilized for the experiments, allowing for enhanced control and standardization in simulating human digestive conditions while preserving bioactive compounds that are sensitive to humidity and temperature during storage. The compounds passed through the oral, gastric, and intestinal phases of digestion before undergoing colonic fermentation for up to 24 h. Throughout this process, the release of polyphenols, their antioxidant activity, and changes in the composition of the microbiota were analyzed. The results indicated that the phenolic compounds associated with the fibers were primarily released during colonic fermentation, which also promoted beneficial changes in the intestinal microbiota and an increase in bacterial genera, suggesting a significant prebiotic effect on gastrointestinal health [79].

Gadicherla et al. (2019) [80] investigated the therapeutic effect of *M. citrifolia* fruit extract for the treatment of L-arginine-induced acute pancreatitis in male rats. The positive control group received melatonin, while the treatment groups received *M. citrifolia* extract at doses of 200 and 400 mg/kg six days before disease induction. Twelve hours after induction, blood samples were collected and demonstrated that administration of *M. citrifolia* fruit extract promoted a significant reduction (*p* < 0.001) in amylase levels (from 330.5 ± 3.23 IU/L in the disease group to 118.8 ± 4.99 IU/L with 400 mg/kg in the extract) and lipase (from 83.17 ± 1.13 U/L to 44.33 ± 1.63 U/L). Histological analysis showed less damage to pancreatic tissue, and DNA assays showed that the extract prevented DNA fragmentation, indicating protection against apoptosis [80].

In the study by Lina et al. (2017) [78], the hepatoprotective effects of naturally fermented noni juice (NJ) against liver fibrosis in rodents were observed. The animals were divided, and fibrosis was induced with thioacetamide. They were subsequently treated with noni juice at different concentrations: low dose (2.51 mL NJ/kg), medium dose (5.02 mL NJ/kg), and high dose (7.52 mL NJ/kg). Finally, it was observed that fermented noni juice showed a protective potential against liver fibrosis through increased antioxidant capacities. Table 7 shows the levels of antioxidant capacity and reduction in liver enzymes in rats [78].

#### 2.4.5. Other Activities

Ratnoglik et al. (2014) [81] evaluated the antidiabetic activity of *M. citrifolia* fruit extract and its bioactive compound, scopoletin, through in vitro enzyme inhibition and glucose uptake assays in HepG2 cells. First, to determine the cytotoxicity of the compounds, cell viability tests were conducted using the 3-(4,5-Dimethyl-2-thiazolyl)-2,5-diphenyl-2H-tetrazolium bromide (MTT), with a concentration of 1 mg/mL defined for the noni extract and 0.2 μM for scopoletin. Subsequently, the potential of the tested compounds to stimulate glucose uptake by HepG2 cells was investigated by measuring the glucose remaining in the medium after treatment. A decrease in glucose levels indicated greater cellular uptake. These experiments demonstrated the potential of noni extract as an aid in controlling blood glucose levels, both by slowing the digestion of carbohydrates and by promoting glucose utilization by the cells [81].

Sasnan, Hanani, and Kristianto (2014) [82] evaluated the effects of *M. citrifolia* extract on individuals with hypercholesterolemia through a double-blind clinical trial. This randomized, placebo-controlled study involved 60 volunteers who were equally divided into a control group and an experimental group. Participants in the experimental group received capsules of noni extract (500 mg, three times a day) for 14 days, while the control group received a placebo, following the same regimen as the experimental group. Blood samples were collected before and after treatment, and the results indicated that, at the end of the trial, the group that consumed the extract experienced a significant reduction in total cholesterol and LDL levels, while the placebo group showed no significant changes. None of the participants reported any adverse effects during the study, suggesting that the use of the extract was safe throughout the trial. The authors concluded that *M. citrifolia* extract may be a promising natural alternative for managing hypercholesterolemia but emphasized the need for further research, particularly long-term studies, to confirm its clinical efficacy and safety with prolonged use [82].

Wan Osman et al. (2019) [83] investigated the therapeutic effects of *Morinda* leaf extract, which is rich in epicatechin and scopoletin, using a monoiodoacetate-induced osteoarthritis model in rats. The animals were divided into control groups and were administered noni extract orally for a duration of 28 days. The results showed that the extract significantly reduced the levels of pro-inflammatory cytokines, such as TNF-α and IL-1β, as well as matrix-degrading enzymes, including MMP-3, in the serum of the treated animals. Furthermore, there was an increase in the levels of endogenous antioxidants, such as glutathione, and a decrease in oxidative stress markers. Histopathological analysis revealed significant preservation of cartilage integrity in the knees of rats treated with noni extract [83].

Moh et al. (2024) [84] investigated the effects of the methanolic extract of *M. citrifolia* fruit on the resistance of *Penaeus vannamei* shrimp to infection by *Vibrio parahaemolyticus*. The post-larval shrimp were fed for 30 days with rations supplemented with varying concentrations of the extract. Following this period, they were challenged with the pathogenic bacteria. The results indicated that the shrimp receiving the highest dosage of the extract exhibited a survival rate increase of 26.7% compared to the control group. Additionally, there was a significant enhancement in the activity of digestive enzymes and antioxidants, along with a reduction in the degeneration of hepatopancreatic cells [84].

Finally, Prompipak et al. (2021) [85] demonstrated that ethanolic extracts of noni fruit combined with 5-fluorouracil (5-FU) exerted a synergistic anticancer effect against cholangiocarcinoma cells both in vitro and in vivo. The combined treatment enhanced apoptosis, increased reactive oxygen species (ROS) production, and significantly reduced tumor growth in xenograft models compared to either treatment alone. While the study did not provide a fractional inhibitory concentration (FIC) index, the quantitative data showed that tumor volume in the combination group decreased by nearly 60% relative to control, whereas 5-FU alone reduced it by 35%. These findings provide strong evidence of the pharmaceutical potential of *M. citrifolia* extracts as adjuvants in combination therapies, supporting the need for further investigations into standardized synergy metrics [85].

### 2.5. Technological Applications

Among the various studies published on *M. citrifolia*, there is a growing interest in its technological applications, particularly in enhancing the bioavailability, stability, and therapeutic efficacy of its bioactive compounds through advanced pharmaceutical formulations and nanotechnology. These innovations aim to address common limitations associated with crude plant extracts, such as instability, poor solubility, and low bioavailability, thereby enabling more effective incorporation of *M. citrifolia* into therapeutic and cosmetic products. Although there are studies on the use of noni in cosmetics, it is not possible to identify the presence of tests on the permeability of its compounds, such as quercetin.

Devanesan et al. (2020) [86] synthesized AgNPs using the fruit extract of *M. citrifolia*. The resulting nanoparticles, with sizes ranging from 12 to 26 nm, displayed strong antioxidant capacity and significant antitumor effects in a murine model of Ehrlich ascites carcinoma, leading to a pronounced reduction in tumor volume and an increase in catalase and superoxide dismutase activities. The study reinforces how green synthesis methods using plant matrices not only offer environmental benefits but also result in functional nanomaterials with biomedical potential [86].

Similarly, Suman et al. (2013) [87] reported the biosynthesis of AgNPs using root extract of *M. citrifolia*, yielding spherical nanoparticles (30–55 nm) that were cytotoxic to HeLa cells. The mechanism was linked to ROS generation, mitochondrial damage, and apoptosis induction [87]. In addition, damnacanthal, an anthraquinone isolated from *M. citrifolia,* demonstrated antiproliferative activity in MCF-7 and HeLa cells through cyclin D1 downregulation and cell cycle arrest at the G1 phase, reinforcing its role as a regulator of tumor cell progression [88]. Recurrent molecular pathways observed in *M. citrifolia* research include oxidative stress induction (ROS), apoptotic signaling (caspase-3 activation, DNA fragmentation), and inhibition of matrix metalloproteinases, all of which contribute to reduced tumor growth. Although cosmetic applications, such as those investigated by Surini and Auliyya (2017) in hydrogel facial masks, expand the plant’s technological reach, the lack of detailed molecular oncology outcomes in such studies reinforces the need for more robust research [89].

In the field of film-based biomaterials, Silva et al. (2021) [90] developed composite films using pectin and/or chitosan matrices enriched with *M. citrifolia* fruit extract. These films demonstrated significantly enhanced antioxidant activity, with DPPH radical inhibition increasing from 63.5% in the pure extract to 84.2% when incorporated into the chitosan–pectin matrix. Additionally, the films showed improved antimicrobial efficacy against Staphylococcus aureus and Escherichia coli. Importantly, the addition of the extract did not compromise the films’ flexibility or structural integrity, although a slight reduction in thermal stability was noted. Overall, the findings support the potential application of these films in advanced wound dressings or food packaging systems, while also emphasizing the need for further optimization to enable industrial-scale production [90].

Another study of interest is by Mohd et al. (2019) [91], which involved the nanoencapsulation of damnacanthal, an anthraquinone compound from *M. citrifolia*, in chitosan particles. The nanoformulation enhanced damnacanthal’s bioavailability and cellular uptake, and its antiproliferative activity via cyclin D1 suppression was superior to that of the free compound. This reinforces the relevance of nanocarriers in enhancing the performance of phytochemicals that would otherwise have limited bioactivity in vivo [91].

In terms of biocompatibility and toxicity, Pawar et al. (2019) [92] explored dermal exposure to gold nanoparticles coated with noni fruit extract in Wistar rats. The absence of significant alterations in hematological or biochemical markers indicated that such nanostructures may be safe for topical administration, though long-term studies remain necessary. Conversely, clinical case reports have described hepatotoxicity associated with noni juice consumption, typically at high doses (>1.5 L/day) or in patients with predisposing factors such as polypharmacy or pre-existing liver conditions. Such events are thought to be linked to anthraquinone content and potential drug–herb interactions, reinforcing the need for careful dose standardization and pharmacovigilance [92]. Lastly, the analgesic potential of the plant was validated in vivo by McKoy et al. (2009) [93], who found that freeze-dried noni fruit powder induced significant antinociceptive effects in mice. The extract also inhibited MMP-9 release, a mediator of inflammatory pain, further supporting its relevance in developing anti-inflammatory formulations [93]. Concerning toxicity, products derived from *M. citrifolia* have a safe genotoxicity profile based on both in vitro and in vivo tests. However, there is a gap in the literature regarding their chronic toxicity in human and animal models. Consequently, additional investigations are needed to assess the safety of their nutraceutical use [94].

### 2.6. Extractivism and Social, Economic, and Cultural Importance

The extraction of *M. citrifolia* in natural environments is typically performed manually, involving the selective harvesting of ripe fruits, which are identified by their whitish coloration and distinct odor [95]. Following collection, the fruits are traditionally subjected to a natural fermentation process in sealed containers over several weeks. This fermentation aims to enhance the preservation of the fruit’s bioactive compounds and is considered integral to traditional preparation methods [96]. Such practices are particularly prevalent in regions like Hawaii and Polynesia, where *M. citrifolia* holds significant cultural and medicinal significance [97]. Regarding the influence of fermentation, Wang et al. (2021) [98] demonstrated that this process substantially alters the phytochemical composition of *M. citrifolia* juice in comparison to its fresh counterpart. Their findings indicated that throughout a 63-day fermentation period, the total phenolic content exhibited dynamic changes, initially declining and subsequently increasing to levels surpassing those observed in fresh juice. Additionally, the flavonoid concentration displayed a consistent upward trajectory during fermentation, increasing from 26.07 mg RE/100 mL in fresh juice to 43.28 mg RE/100 mL at an industrial scale, thereby augmenting the juice’s antioxidant capacity [98].

Furthermore, regarding its economic significance, the widespread distribution of *M. citrifolia* in tropical climates, particularly in countries such as the United States, Brazil, and Tahiti, as well as in regions of East Asia and Australia, has contributed significantly to the plant’s commercial potential. In commercially available formulations, the levels of bioactive compounds found are lower, making their effect on health uncertain. In this regard, it is interesting to note that factors such as the lack of standardized analytical methods and the robustness of clinical results make it difficult to assess the efficacy of commercial doses [99,100]. This is largely attributed to variations in harvesting conditions and diverse phytochemical profiles. Different parts of the plant, including its fruits, seeds, bark, leaves, and flowers, are utilized separately for both nutritional and therapeutic purposes [101]. *M. citrifolia* gained popularity as a health supplement within the first twelve years of its commercial introduction and is now consumed in over 80 countries. In recent years, the fruit has increasingly been used to produce dietary supplements, with noni fruit purée becoming one of the primary agricultural exports of French Polynesia. In that region, the plant is also traditionally employed to treat osteoarthritis, rheumatism, and back pain [102].

Regarding its social and cultural significance, as previously mentioned, *M. citrifolia* is important in the traditional medicine of the Polynesian people, who inhabit the South Pacific islands, including Tonga, Tuvalu, French Polynesia, the Cook Islands, Niue, Easter Island, and Hawaii. The Polynesians utilize the entire noni plant in their medicinal remedies and as dyes for their traditional clothing. The roots, stems, bark, leaves, flowers, and fruits of the noni plant are all incorporated in various combinations in nearly 40 recognized herbal remedies. Consequently, the prominence of noni is evident both culturally and socially, as it has been intertwined with the heritage of these communities for thousands of years [103].

### 2.7. Conservation of Morinda Citrifolia

*M. citrifolia* is a significant resource in the traditional medicine of societies in the Pacific Islands. Its resilience allows it to thrive in poor, saline soils and in areas subjected to environmental stressors such as wind, fire, and floods, which contributes to its considerable social and economic importance. However, despite its ability to survive in diverse environmental conditions, the plant is susceptible to a wide range of pests and disease-causing pathogens, especially when cultivated in a modern monoculture agricultural system [97].

Therefore, agroforestry practices that incorporate noni not only help to reduce pressure on native forests but also provide an economically viable and environmentally sustainable alternative for land use and plant preservation. Unlike monocultures, agroforestry plantations enhance genetic diversity, which reduces the likelihood of large pest infestations destroying the entire crop [104]. Furthermore, it is also interesting to mention that ex situ conservation techniques, such as in vitro micropropagation, offer viable alternatives to produce healthy seedlings and the preservation of germplasm, as demonstrated by Shekhawat et al. (2015) [105].

## 3. Methodology

### Search in Databases and Inclusion and Exclusion Criteria

The keyword “*Morinda citrifolia*” was used as the main index for searching the following databases: PubMed^®^, SciElo, Scopus^®^, and Web of Science^®^. Articles published in English, Spanish, and Portuguese, and without restriction on year of publication, were considered. Articles published in conference proceedings, course completion papers, dissertations, and theses, and those without mention of the ethnomedicinal uses, biological activities, phytochemistry, conservation, or technological applications of *M. citrifolia* were discarded.

## 4. Conclusion and Perspectives

Despite studies supporting the therapeutic potential of *M. citrifolia* L., significant knowledge gaps still hinder its integration into evidence-based clinical practice. Most investigations remain limited to experimental models, while well-designed and controlled clinical trials, crucial for establishing efficacy and safety in humans, are still scarce. Furthermore, the lack of standardized extraction methods and the incomplete identification of the bioactive compounds responsible for its diverse pharmacological activities represent persistent challenges. Notably, the species’ antioxidant and fungicidal properties stand out as particularly promising. Although these effects have been consistently demonstrated, they still require clinical validation. Advancing research in these directions would not only reinforce the therapeutic value of *M. citrifolia* but also expand its potential applications in innovative pharmaceuticals, nutraceuticals, and cosmetics. Therefore, future studies should prioritize robust clinical trials, the development of extract standardization protocols, and investigations that unravel the isolated and synergistic contributions of their active constituents.

## Figures and Tables

**Table 1:** Phytochemical profile of *Morinda citrifolia*: compound structures, extract types, and plant part origin.

Compound	Compound Structure	Extract Type	Plant Source
Anthraquinones			
Morindicone (9-hydroxy-2-methoxy-4-methyl-3,10-anthracenedione)	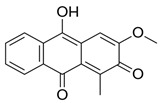	Methanolic extract, fractioned with ethyl acetate, ether, and *n*-hexane	Stem
1-Hydroxy-2-methylanthraquinone	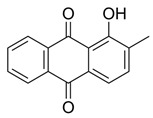	Methanolic extract, fractioned with ethyl acetate, ether, and *n*-hexane	Stem
2-Hydroxymethylanthraquinone	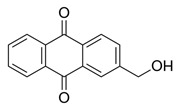	Methanolic extract, fractioned with ethyl acetate, ether, and *n*-hexane	Stem
1,3,5-Trihydroxy-2-methoxy-6-(methoxymethyl)-9,10-anthracenedione	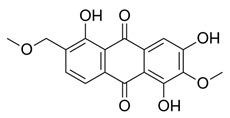	Ethanolic extract (90%), fractioned with petroleum ether and ethyl acetate	Fruit
1,3,5-Trihydroxy-2-methoxy 6-(methoxymethyl)-9,10-anthracenedione	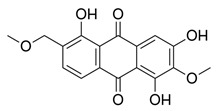	Ethanolic extract (90%), fractioned with petroleum ether and ethyl acetate	Fruit
3-Hydroxy-1,2,4-trimethoxy-6-methylanthraquinone	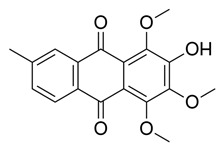	Ethanolic extract (90%), fractioned with petroleum ether and ethyl acetate	Fruit
Xanthones			
Morinthone (4-methoxy-3-heptadecylxanthone)	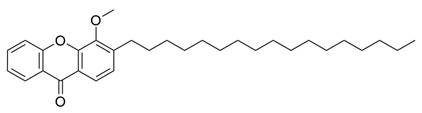	Methanolic extract, fractioned with ethyl acetate, ether, and *n*-hexane	Stem
Plocamanone C	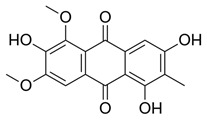	Ethanolic extract (90%), fractioned with petroleum ether and ethyl acetate	Fruit
Iridoid Glycosides			
Asperuloside	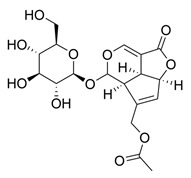	Methanolic extract, partitioned with *n*-butanol	Fruit (dehydrated and pulverized)
Asperulosidic acid	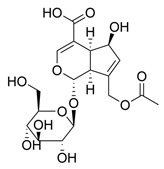	Methanolic extract, partitioned with *n*-butanol	Fruit (dehydrated and pulverized)
Geniposidic acid	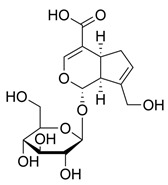	Methanolic extract, sequential partitioning	Fruit
Deacetylasperulosidic acid	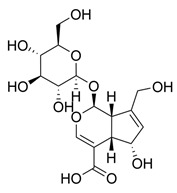	Methanolic extract, sequential partitioning	Fruit
Fatty Acid Esters			
Methyl linoleate	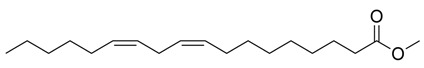	Methanolic extract, fraction EMF1	Seed
Methyl palmitate	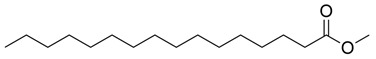	Methanolic extract, fraction EMF1	Seed
Methyl oleate	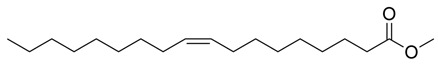	Methanolic extract, fraction EMF1	Seed
Methyl stearate	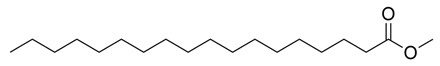	Methanolic extract, fraction EMF1	Seed
Glycosides			
Nonioside D	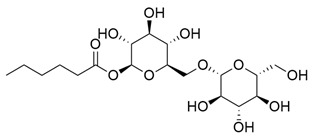	Methanolic extract, sequential partitioning	Fruit
Nonioside F	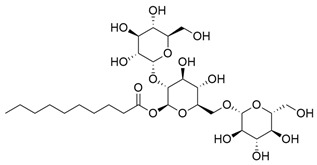	Methanolic extract, sequential partitioning	Fruit
Nonioside C	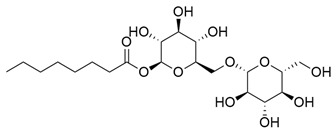	Methanolic extract, sequential partitioning	Fruit
Hemiterpene Glycosides			
Nonioside A	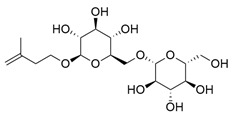	Methanolic extract, sequential partitioning	Fruit
3-methylbut-3-en-1-yl-β-D-glucopyranoside	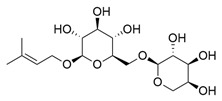	Methanolic extract, sequential partitioning	Fruit
γ-Lactones			
5-Butyloxolan-2-one	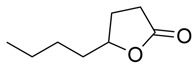	Methanolic extract, fraction EMF1	Seed
5-Hexyloxolan-2-one	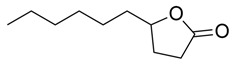	Methanolic extract, fraction EMF1	Seed
Alkene			
(E)-Octacos-2-ene	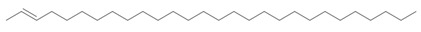	Methanolic extract, fraction EMF1	Seed
Triterpenes			
Ursolic acid	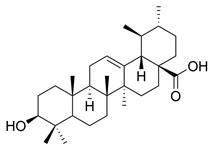	Methanolic extract, sequential partitioning	Fruit
Oleanolic acid	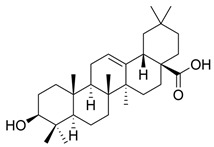	Methanolic extract, sequential partitioning	Fruit
Sterols			
β-Sitosterol	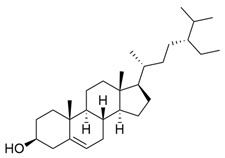	Methanolic extract, sequential partitioning	Fruit
Stigmasterol	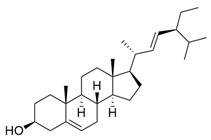	Methanolic extract, sequential partitioning	Fruit
β-Sitosterol 3-O-β-D-glucopyranoside	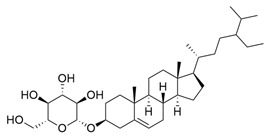	Methanolic extract, partitioned with *n*-butanol	Fruit (dehydrated and pulverized)
Alkaloids			
Borreriagenin	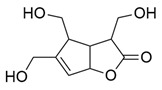	Methanolic extract, partitioned with *n*-butanol	Fruit (dehydrated and pulverized)
Alcohol			
D-Mannitol	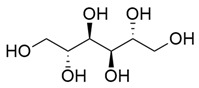	Methanolic extract, partitioned with *n*-butanol	Fruit (dehydrated and pulverized)
Nucleoside			
Cytidine	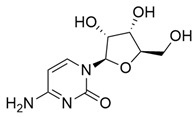	Methanolic extract, partitioned with *n*-butanol	Fruit (dehydrated and pulverized)
Neolignan			
Americanin A	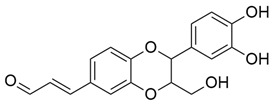	Methanolic extract, partitioned with *n*-butanol	Fruit (dehydrated and pulverized) and Seed
Phenolic Acid			
Vanillic acid	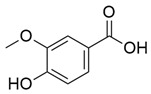	Methanolic extract, sequential partitioning	Fruit
Coumarin			
Scopoletin	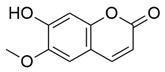	Methanolic extract, fraction EMF2	Seed
Terpenoids			
(+)-Austrosene	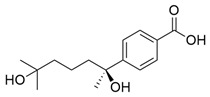	Ethanolic extract (90%), petroleum ether fraction	Stem and Leaves
(6S)-2-Methyl-6-(4-formylphenyl)-2-hepten-4-one	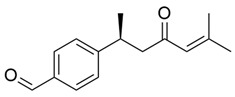	Ethanolic extract (90%), petroleum ether fraction	Stem and Leaves
Asperterpenoid A	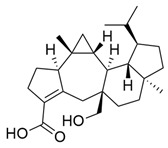	Ethanolic extract (90%), petroleum ether fraction	Stem and Leaves

**Table 2:** Comparative distribution of Anthraquinones in *Morinda citrifolia*.

Plant Part	Relative Quality of Anthraquinones	Main Identified Compounds
Roots	Very high	Damnacanthal, nordamnacanthal, morindone, derivatives
Stem	Moderate	Various anthraquinone derivatives
Branches	Trace amounts	Minor anthraquinone peaks, non-significant
Leaves	Low	Diverse compounds with different polarities
Fruits	Very Low	2-methoxy-1,3,6-trihydroxyanthra quinone and other minor compounds
Cell culture	Lower than roots, variable	Anthraquinones produced in vitro, but at reduced levels

**Source:** Shami (2015) and Wang et al. (2019) [39,40].

**Table 3:** IC_50_ of isolated compounds from *Morinda citrifolia*.

Isolated Compound	Plant Fragment	Evaluated Activity	IC_50_ (μM)
Americanin	Fruit	Antioxidant (DPPH)	1.8
Lignan 1 (not named)	Fruit (Tahiti)	5-lipoxygenase inhibitor	0.43
Lignan 2 (not named)	Fruit (Tahiti)	15-lipoxygenase inhibitor	1.0
Scopoletin	Fruit	Antioxidant (DPPH)	9.5
Compound A-D (glycosides)	Fruit	Glucuronidase inhibitor	0.62 to 6.91

**Abbreviations:** 1,1-diphenyl-2-picrylhydrazyl (DPPH); Half Maximal Inhibitory Concentration (IC_50_) **Source:** Su et al. (2005); Kim et al. (2020) [37,53].

**Table 4:** IC_50_ of *Morinda citrifolia* extracts.

Extract	Plant Fragment	Evaluated Activity	IC_50_
Fresh juice	Fruit	Antioxidant (DPPH)	0.024 mg/mL
Fermented juice	Fruit	Antioxidant (DPPH)	0.047 mg/mL
Methanol/Acetone	Fruit pulp	Antioxidant (DPPH)	25.18 mg/ mL
Methanolic	Fruit pulp	Antioxidant (DPPH)	25.96 mg/mL
Ethanol	Fruit pulp	Antioxidant (DPPH)	40.98 μg/mL
Ethanol	Fruit pulp	Antioxidant (DPPH)	103.2 μg/mL
Acetonic	Fruit peel	Antioxidant (DPPH)	105.79 μg/mL
Acetonic	Seed	Antioxidant (DPPH)	108.19 μg/mL
Aqueous	Fruit pulp	Antioxidant (DPPH)	1.402 μg/mL
Ethanol (75%)	Sheet	Antioxidant (DPPH)	4.27 g/L
Ethyl acetate	Seed	Antioxidant (TLC-DPPH)	0.7 μg/mL
Gross	Fruit	*L. amazonensis*	63.6 μg/mL

**Abbreviations:** Thin-layer chromatography (TLC) with 1,1-diphenyl-2-picrylhydrazyl (DPPH); Half Maximal Inhibitory Concentration (IC_50_). **Source:** Sina et al. (2020) [29].

**Table 5:** Minimum inhibitory concentrations of isolated compounds with antimicrobial activity.

Isolated Compounds	Plant Fragment	Target Microorganism	MIC (μM)
Scopoletin	Fruit	*Staphylococcus aureus*	35.5
Damnacanthal	Root	*Escherichia coli*	12.3
Octanoic acid	Fruit	*Candida albicans*	45.0
Morindona	Root	*Pseudomonas aeruginosa*	30.7
Ursolic acid	Sheet	*Salmonella typhimurim*	28.1
Rubiadin	Root	*Klebsiella pneumoniae*	50.2

**Abbreviations:** Minimum inhibitory concentration (MIC).

**Table 6:** Minimum inhibitory concentrations of *Morinda citrifolia* extracts with antimicrobial activity.

Isolated Compounds	Plant Fragment	Target Microorganism	MIC (μg/mL)
Ethanol	Ripe fruit	*Staphylococcus aureus*	85.2
Aqueous	Sheet	*Escherichia coli*	120.5
Chloroform	Ripe fruit	*Pseudomonas aeruginosa*	60.8
Ethyl acetate	Sheet	*Candida albicans*	45.0
Methanolic	Roots	*Salmonella typhimurim*	70.3
Hexane	Stem bark	*Klebsiella pneumoniae*	95.6

**Abbreviations:** Minimum inhibitory concentration (MIC).

**Table 7:** Effect of *Morinda citrifolia* on liver biomarkers and antioxidant enzymes.

Biomarker	TAA (disease)	TAA + NJ-L	TAA + NJ-L	TAA + NJ-H
ALT	-	−20.01%	−29.2%	−35.7%
AST	-	−45.72%	−55.26%	−56.65%
TBARS	0.28 ± 0.01	0.26 ± 0.01	0.26 ± 0.01	0.25 ± 0.01
SOD	19.23 ± 1.80	25.00 ± 0.92	26.86 ± 1.29	28.70 ± 2.82
CAT	20.47 ± 0.70	25.24 ± 1.31	29.57 ± 0.97	28.73 ± 0.90
GSH-Px	51.01 ± 2.19	70.85 ± 3.08	73.71 ± 2.83	79.19 ± 1.36

**Abbreviations:** ALT: alanine aminotransferase; AST: aspartate aminotransferase; TBARS: thiobarbituric acid reative substances (n mole MDA eq./mg protein); MDA: malondialdehyde; SOD: superoxide dismutase (munit/mg protein); CAT: catalase (unit/mg protein); GSH-Px: glutathione peroxidase (nmole NADPH oxidized/min/mg protein); TAA: thioacetamide; NJ: Noni juice; L/M/H: low, medium, high doses. **Source:** Lin et al. (2017) [78].

## Data Availability

Data is included within this article.

## Abbreviations

ABTS	2,2′-azino-bis(3-ethylbenzothiazoline-6-sulfonic acid)
AgNPs	Silver nanoparticles
DPPH	1,1-diphenyl-2-picrylhydrazyl
FRAP	Ferric reducing antioxidant power
GAE	Gallic acid equivalent
IC_50_	Inhibitory concentration
IL-6	Interleukin-6
*M. citrifolia*	*Morinda citrifolia*
MIC	Minimum inhibitory concentration
MRSA	Methicillin-resistant *Staphylococcus aureus*
MTT	3-(4,5-dimethyl-2-thiazolyl)-2,5-diphenyl-2H-tetrazolium bromide
QE	quercetin equivalents
RNS	Reactive nitrogen species
ROS	Reactive oxygen species
*S. epidermidis*	*Staphylococcus epidermidis*
*S. haemolyticus*	*Staphylococcus haemolyticus*
TLC	Thin-layer chromatography
TNF-α	Tumor necrosis factor alpha
